# Superlattice-based thin-film thermoelectric modules with high cooling fluxes

**DOI:** 10.1038/ncomms10302

**Published:** 2016-01-13

**Authors:** Gary Bulman, Phil Barletta, Jay Lewis, Nicholas Baldasaro, Michael Manno, Avram Bar-Cohen, Bao Yang

**Affiliations:** 1 RTI International, Electronics and Applied Physics Division, Research Triangle Park, North Carolina 27709, USA; 2 Department of Mechanical Engineering, University of Maryland, College Park, Maryland 20742, USA

## Abstract

In present-day high-performance electronic components, the generated heat loads result in unacceptably high junction temperatures and reduced component lifetimes. Thermoelectric modules can, in principle, enhance heat removal and reduce the temperatures of such electronic devices. However, state-of-the-art bulk thermoelectric modules have a maximum cooling flux *q*_max_ of only about 10 W cm^−2^, while state-of-the art commercial thin-film modules have a *q*_max_ <100 W cm^−2^. Such flux values are insufficient for thermal management of modern high-power devices. Here we show that cooling fluxes of 258 W cm^−2^ can be achieved in thin-film Bi_2_Te_3_-based superlattice thermoelectric modules. These devices utilize a p-type Sb_2_Te_3_/Bi_2_Te_3_ superlattice and n-type δ-doped Bi_2_Te_3−*x*_Se_*x*_, both of which are grown heteroepitaxially using metalorganic chemical vapour deposition. We anticipate that the demonstration of these high-cooling-flux modules will have far-reaching impacts in diverse applications, such as advanced computer processors, radio-frequency power devices, quantum cascade lasers and DNA micro-arrays.

## Abstract (web summary)

Current thermoelectric modules provide cooling fluxes that are insufficient for high-heat flux applications. Here, the authors demonstrate thin-film-based thermoelectric modules capable of providing cooling fluxes more than double that of the current state-of-the-art.

## Graphical abstract

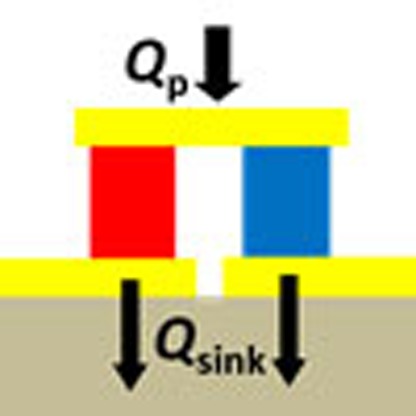

Thermoelectric modules have long been considered for cooling high-power electronic^1^,^2^,^3^,^4^,^5^ and opto-electronic devices^6^, and there have been consistent efforts to improve the cooling flux values of thermoelectric modules in the past decade^7^,^8^,^9^,^10^,^11^,^12^,^13^,^14^,^15^. The maximum cooling flux *q*_max_ of a thermoelectric module is defined as the maximum cooling flux that the thermoelectric module is capable of providing at a temperature difference across the module (Δ*T*) of zero and is given by equation (1)^16^,^17^ below.


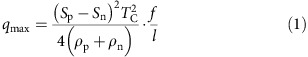


where *S*_p_ and *S*_n_ are the Seebeck coefficients of the p- and n-type elements, respectively, *ρ*_p_ and *ρ*_n_ are the electrical resistivities of the p- and n-type elements, respectively, *T*_C_ is the temperature of the cold side of the thermoelectric module, *f* is the packing fraction and *l* is the thickness of the thermoelectric elements. Equation (1) shows that *q*_max_ of the thermoelectric module depends on the thermoelectric material properties, the element thickness and the packing fraction.

One approach for increasing *q*_max_ is to develop thermoelectric materials with a high thermoelectric figure of merit ZT (refs 1, 7, 8, 9, 10, 11, 12, 13, 14, 15, 18, 19, 20, 21, 22, 23, 24), as defined in equation (2).


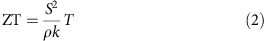


where *S*, *ρ*, *k* and *T* are the Seebeck coefficient, electrical resistivity, thermal conductivity and absolute temperature, respectively. Significant progress has been made in recent years to increase ZT using nanostructured materials such as thin-film superlattices^1^,^18^,^25^,^26^, thick films of quantum-dot superlattices^14^ and nanocomposites^7^,^8^,^9^.

Alternatively, *q*_max_ of the thermoelectric module can be increased by reducing the element thickness, as *q*_max_ is inversely proportional to *l* (refs 1, 18, 26). However, the reduction of element thickness is limited by two factors: the synthesis method for the thermoelectric elements; and the electrical contact resistance between the thermoelectric elements and the copper trace. Electrical constant resistance has a marked impact on the performance of thin thermoelectric modules since the magnitude of the contact resistance can be comparable to that of thermoelectric element itself. Bulk thermoelectric materials cannot be thinned below a few hundred microns, resulting in the modest maximum cooling flux of approximately 10 W cm^−2^ (refs 27, 28). Epitaxial semiconductor films can be grown much thinner, resulting in a higher maximum cooling flux for thin-film modules. As a point of reference, commercially available thin-film thermoelectric modules have elements that are approximately 20-μm thick and have maximum cooling fluxes around 100 W cm^−2^ (ref. 29). However, even the cooling flux of state-of-the art thermoelectric modules is insufficient for advanced thermal management application. For instance, heat generation in a silicon microprocessor is non-uniform, with localized heat flux possibly larger than 200 W cm^−2^, and GaN-based transistors can produce hotspots with heat fluxes in excess of 1 kW cm^−2^ (refs 4, 30, 31).

In this paper, we present thermoelectric module capable of producing a cooling flux of 258 W cm^−2^, more than double that of the current state-of-the-art value. The enhancement in the module-level cooling flux is a result of thin (8.1 μm) Bi_2_Te_3_-based thin-film superlattice materials with high intrinsic ZT, the δ-doped n-type structure, and reduced electrical contact resistances (2.68 × 10^−7^ Ω cm^2^ for n-type and 1.36 × 10^−6^ Ω cm^2^ for p-type).

## Results

### Material synthesis and module fabrication

**Figure 1:**
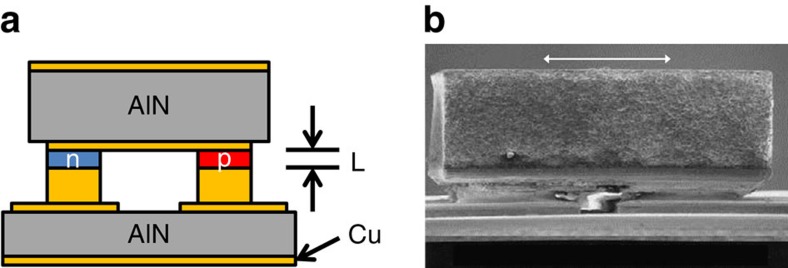
Cross-sectional views of a Bi_2_Te_3_-based thin-film thermoelectric module. (**a**) Illustration of thin-film-based thermoelectric module, showing top and bottom AlN headers, Cu traces and n-type and p-type active elements. *L* represents the length of the active elements, which is 8 μm in the present work. Figure is not to scale. (**b**) Scanning electron microscope image of the upper portion of a completed thin-film superlattice thermoelectric module. Scale bar, 250 μm.

Figure 1a,b show the structure of the thermoelectric module. The thermoelectric material at the heart of the module consists of p-type 10/50 Å Bi_2_Te_3_/Sb_2_Te_3_ superlattice and n-type δ-doped Bi_2_Te_3−*x*_Se_*x*_, both of which are grown heteroepitaxially using metalorganic chemical vapour deposition^18^. The n-type structure is grown by periodically interrupting the growth of Bi_2_Te_3−*x*_Se_*x*_ and dosing the flow with Te and Se species. This δ-doping process can result in an increase in carrier concentration without a reduction in electron mobility. In the present experiment, 8.1-μm-thick thin films are grown and fabricated into cooling devices.

The ZT values of the Bi_2_Te_3_/Sb_2_Te_3_ superlattice materials used in this study have been previously measured by two different methods. One is the direct measurement of ZT by Harman method, which reported ZT>2 in ref. 18. The second method is the determination of individual thermoelectric properties, such as Seebeck coefficient, electrical resistivity and thermal conductivity, followed by calculation of ZT. The ZT values of representative p- and n-type materials used in the devices in the present study were estimated, using this indirect technique, to be 1.4 and 1.5, respectively. These data have been added in Table 1. These values were determined through measurement of the in-plane electrical resistivity and Seebeck coefficient of representative material along with an estimation of thermal conductivity via an in-couple-property-validation model. Details of this model are given in ref. 32. Both direct measurement and indirect measurement of ZT were conducted at *T*=300 K. These estimated ZT values by the indirect method are slightly less than the ZT data measured directly via the Harman method for similar materials in ref. 18.

In the device fabrication process, the top epitaxial surfaces of the p- and n-type materials are first metalized and then bonded to a common metalized AlN die header. Following chemical removal of the substrate, electrical contacts are fabricated on the exposed surface (typically with two n- and two p-type circular contacts designated as the 2N–2P configuration). This die header subassembly is then inverted and bonded to a second AlN header that contains the electrical traces used to power the module. As shown in Fig. 1b, the basic completed module structure has a 600 × 600-μm^2^ top header area and is 550 μm tall.

### Electrical contact resistance

One significant barrier to reaching high cooling fluxes with thin-film thermoelectric modules is electrical contact resistance between the metal electrodes and the thermoelectric elements, especially for Bi_2_Te_3_-based materials with low intrinsic electrical resistivity (10^−3^ Ω cm)^1^,^26^. In the present experiment, two metallization methods (plated Au and evaporated Cr/Ni/Au metallization) and two different superlattice structures (standard structures and δ-doped structures) are explored to improve the electrical contact resistance. Au diffusion into the Bi_2_Te_3_ lattice may very well be happening in the thermoelectric devices with plated Au contacts. The detailed effects of this potential Au diffusion need further study in the future.

**Figure 2:**
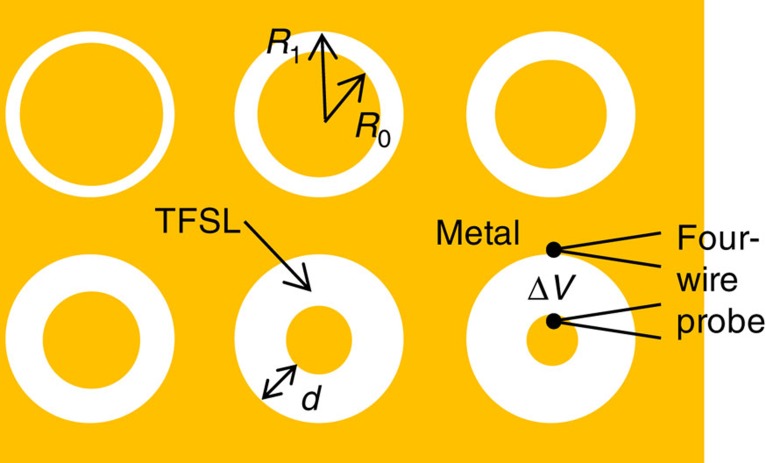
Example of circular TLM patterns used for contact resistivity determination. In this measurement technique, six gaps of increasing widths (*d*=*R*_1_−*R*_0_) are patterned onto the sample surface. The gold colour represents the metal contact, while the white is the thin-film superlattice (TFSL) surface. A four-wire probe is used to apply a small current across each gap and measure the voltage drop (Δ*V*).

Electrical contact resistivity has been measured using the transmission line measurement technique^33^, which measures the resistance across an annular gap as a function of gap width, fabricated on a broad area of metallization on the top surface of the thin-film superlattice material. The metalized contacts are formed on the thin-film superlattice surface as shown in Fig. 2, through the use of photoresist masks (gold is the metal contact, white is the thin-film superlattice surface) to form a set of six gaps with different gap widths. A four-wire probe is used to apply a small current across each gap (with two of the probe wires) and measure the voltage drop across the thin-film superlattice gap (with the other two probe wires). The gap resistance *R*_g_ (measured Δ*V* over current) is a function of the gap width, governed by the relationship shown in equation (3).


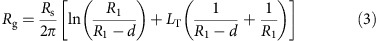


The gap resistance *R*_g_ versus gap width *d* data is fitted to equation (3), with *R*_s_ (sheet resistance) and *L*_T_ (transfer length) as fitting parameters. Contact resistivity *ρ*_c_ can then be calculated as *R*_s_ × *L*_T_^2^ with units of Ω m^2^. In the present modules, plated Au was used as the contact to the source (top) side of the n-type δ-doped Bi_2_Te_3−*x*_Se_*x*_, while evaporated Cr/Ni/Au was used as the source (top) side of the p-type Sb_2_Te_3_/Bi_2_Te_3_. Plated Au was used as the sink (bottom) side contact of both the n- and p-type elements. Electric contact resistivity for thin-film superlattice materials and selected contact metals is shown in Table 2. The *R*_s_, *L*_T_ and *ρ*_c_ values given in Table 2 are an average of the 6–8 experimental values measured for each material type and metallization scheme. The s.d. among these measurements is provided as well.

### Maximum temperature difference Δ*T*_max_

To evaluate the maximum temperature difference Δ*T*_max_, the thermoelectric module is placed directly on a water-cooled heat sink, maintained at 25 °C, and the *T*_C_ and *T*_H_ values are measured using 25 μm diameter thermocouples as a function of current supplied to the module, *I*. The voltage *V* is also monitored as a function of electric current under vacuum (pressure, *P*<1 mTorr), up to the current producing the maximum Δ*T*_max_, which defines the current value of *I*_max_.

The measured temperature difference and voltage behaviour of the thermoelectric module are given by^28^,^34^:


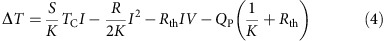



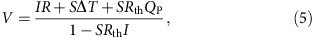


**Figure 3:**
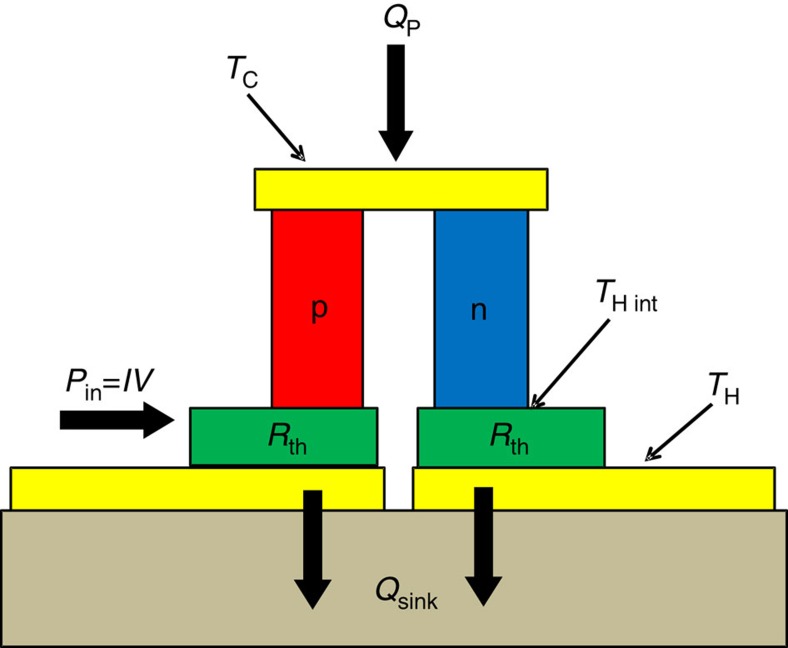
Thin-film thermoelectric module structure. The red and blue rectangles represent the p- and n-type active TE materials, respectively. Yellow represents the current traces and tan represents the ceramic bottom header of the module. Heat entering the module is given by *Q*_P_, while the heat being pumped out is given by *Q*_sink_. *T*_C_, *T*_H_ and *T*_H int_ are the cold-side temperature, externally measured hot-side temperature and internal element hot-side temperature, respectively. The green rectangles represent the parasitic thermal resistances *R*_th_, which reduce the externally observed Δ*T* value. Figure is not drawn to scale.

In the above equations, *S* is the module Seebeck coefficient, which is determined by the voltage between the two electrical leads, *V*, and the temperature difference between the two AIN headers, Δ*T*. The module electric resistance is denoted by *R*, and consists of the electric resistance of the n and p elements *R*_element_, the electric resistance of the metal traces *R*_trace_, and the electric contact resistance between the elements and the metal traces *R*_contact_. The module electric resistance *R* can be determined by the voltage between the two electrical leads, *V*, and the electrical current *I* through the electrical leads. *K* is the module thermal conductance between the two AIN headers. *R*_th_ is the parasitic thermal resistance of the module, which is the difference between the module thermal resistance and the thermal resistance of the n and p thermoelectric element pair (Fig. 3). In the Δ*T*_max_ measurements, there is no heat pumped by the module (cooling power, *Q*_P_=0) and the module parameter values can be determined by fitting equation (4) to the experimental Δ*T* as a function of *I*, *V*, *T*_C_ and *T*_H_ data, and similarly fitting equation (5) to the voltage data. The inclusion of a parasitic thermal resistance *R*_th_ is necessary when considering thin-film thermoelectric modules, because the high-heat fluxes that occur within the module produce an internal element hot-side temperature *T*_H int_ that is different from the externally measured value *T*_H_.

**Figure 4:**
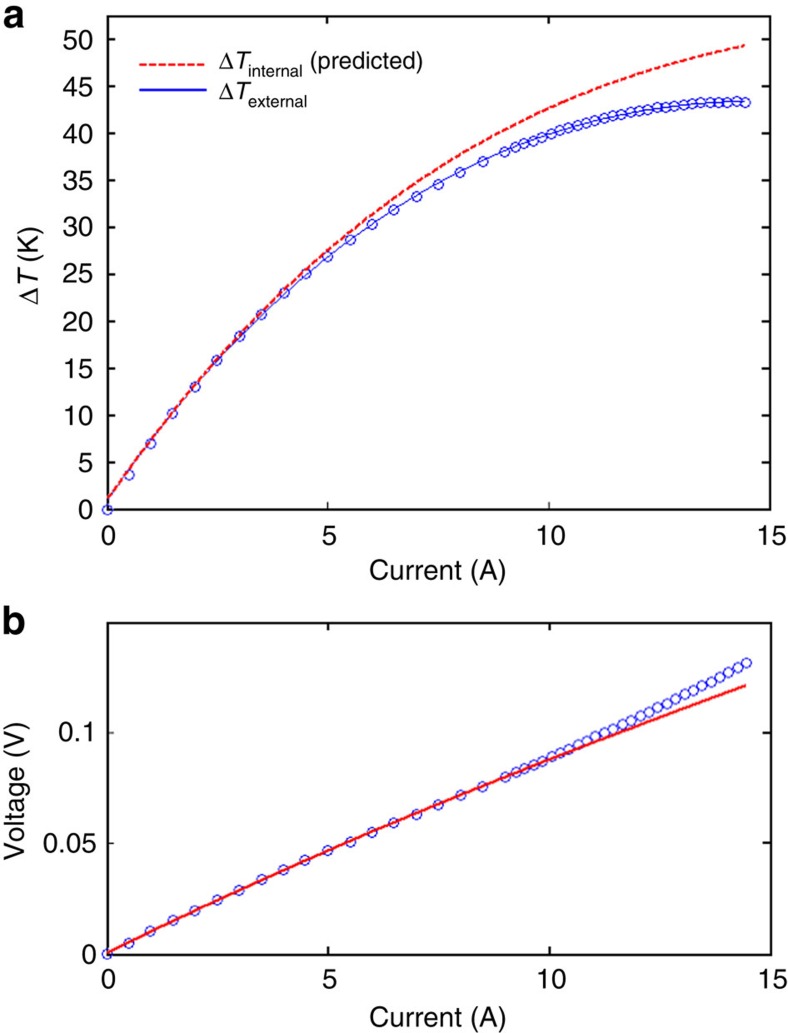
Analysis of experimental data. (**a**) Experimental Δ*T* versus *I* data for the module with the largest effective contact diameter (230 μm) and the fit of equation 4 to the data. Blue dots represent experimentally measured data points, and the blue line represents the fit . The red dotted line indicates predicted internal temperature difference (*T*_hint_−*T*_C_) versus *I*. (**b**) Corresponding *V* versus I data for the same module. The blue dots represent experimentally measured data points, and the red line represents the theoretical fit.

The upper portion of Fig. 4 shows Δ*T* versus *I* data for the module with the largest contact diameter (230 μm). The circles indicate the experimental external Δ*T* values measured with thermocouples, and the curve drawn through these points is the fit of equation (4) to the data, using a parasitic thermal resistance of *R*_th_*=*(3.08±1.98) K W^−1^. An external Δ*T*_max_ value of 43.54 K is observed at an electric current of 14.8A, and the upper dashed curve shows the predicted internal Δ*T* (*T*_H int_−*T*_C_) occurring inside the module, which has a maximum value of 49.3, 5.7 K higher than the external value. The difference in internal and external Δ*T* is caused by the internal thermal resistance *R*_th_, which reduces the external Δ*T* that can be measured by the thermocouples. The multidimensional fit of equation (4) to experimental Δ*T*_external_ versus *I* data also yields the values of the ratios *S*/*K* (0.0228 μV W^−1^) and *R*/*K* (0.349 Ω K W^−1^).

Figure 4b shows the voltage versus current data for the same module. *R* can be determined via a one-dimensional least squares fit of equation (4) to this experimental *V* versus *I* data, which subsequently allows for the calculation of *K* and *S* from the previously determined *R*/*K* and *S*/*K* values. The resulting values of the total Seebeck coefficient *S=*(450±48) μV K^−1^, thermal conductance *K=*(19.7±2.1) mW K^−1^ and electric resistance *R=*(6.87±0.01) mΩ are given in the legend of Fig. 4b. Details of this procedure have been described elsewhere^34^.

### Maximum cooling flux *q*_max_

The cooling power for a thermoelectric module in the presence of a parasitic thermal resistance *R*_th_ is shown in equation (6), where Δ*T* is the externally measured temperature difference^28^,^34^.


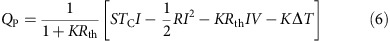


In the case of *R*_th_=0, equation (6) reduces to the standard thermoelectric heat-pumping equation^35^, where the maximum cooling power *Q*_P_ occurs when *I=ST*_C_*/R* (which is *I*_max_). To determine *I*_max_ in the case of non-zero *R*_th_, the voltage dependence in equation (6) is eliminated using equation (5), and after some rearrangement the expression for *Q*_P_ becomes:


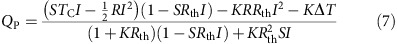


**Figure 5:**
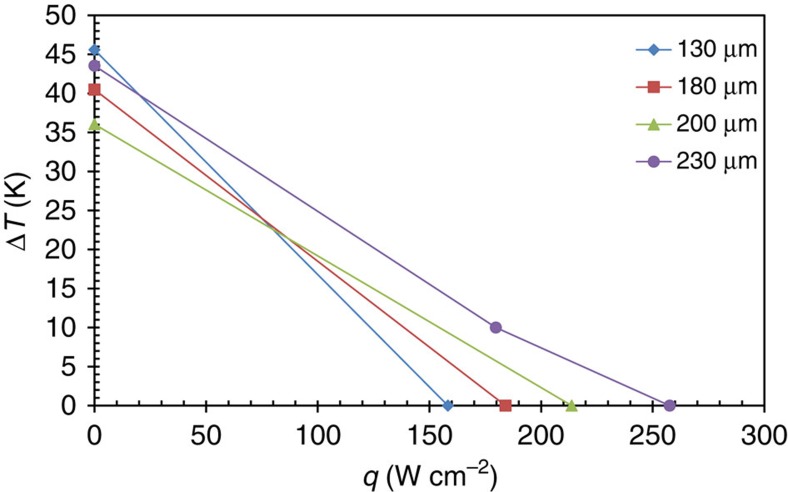
Load lines for each of the four measured modules of this work. The load lines are determined by the measurement of *q*_max_ at Δ*T*=0K, and the measurement of Δ*T*_max_ at *q*=0 W m^−2^. An additional data point for the case of Δ*T*=10 °C is included for the module with the largest contact diameter (230 μm).

Equation (7) is then differentiated with respect to *I* and solved numerically for the value of *I* that yields a zero of the resulting expression. The *I*_max_ value is then substituted into equation (7), along with the individual fitted module parameter values to obtain the *Q*_max_ value that is expected. The *I*_max_ values calculated using equation (5) are found to agree with the experimentally observed values in these modules and are smaller than what is obtained from using the standard expression *I*_max_*=ST*_C_*/R*. The maximum cooling flux *q*_max_ is calculated by dividing the total cooling power *Q*_max_ by the top header area of the module, which is 600 × 600 μm^2^. The performance of four thin-film thermeoelctric modules with different contact diameters (that is, the packing fraction) is summarized in Table 3. The table shows the measured maximum cooling flux (*q*_max_*=Q*_max_*/A*_T_), as well as the predicted value and a discrepancy of 13% is observed between the predicted and measured values. The coefficient of performance (COP) values were calculated at *q*_max_; that is for Δ*T=*0 K. With the establishment of the *q*_max_ value and Δ*T* values, the load lines can be determined, as shown in Fig. 5.

In the case of the largest module, an additional data point that was obtained at Δ*T=*10 °C is also shown. The maximum temperature difference Δ*T*_max_ should not be dependent on the module geometry, but the variation seen indicates a possible undetermined parasitic phenomenon may be present. Further studies are needed to identify this undetermined parasitic phenomenon.

It can be seen that a maximum cooling flux in excess of 250 W cm^−2^ is achieved in the thin-film Bi_2_Te_3_ superlattice thermoelectric module with a contact diameter of 230 μm (that is, 48% packing fraction). This value is 25 times higher than is typically observed in commercial-off-the-shelf bulk thermoelectric modules (http://www.marlow.com) and more than 2.5 times better than commercial-off-the-shelf thin-film modules (http://www.lairdtech.com). The maximum cooling flux of the module was measured using two different methods, the Q-meter method by RTI and the non-contact IR method by University of Maryland.

### Contact diameter and packing fraction

**Figure 6:**
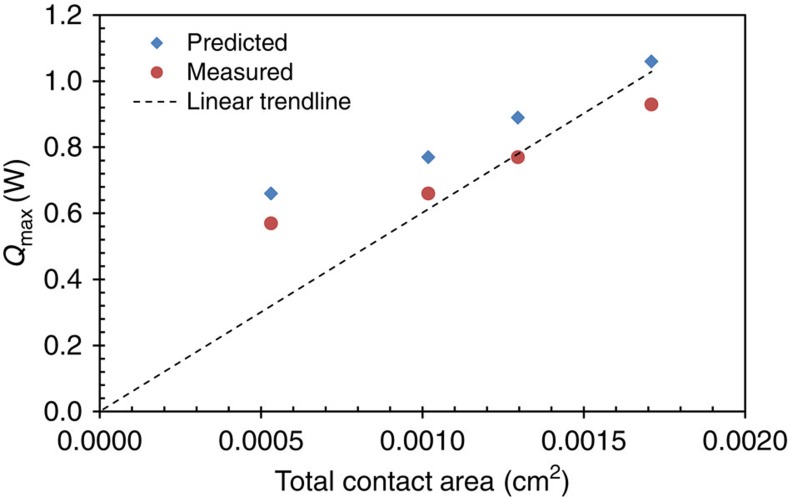
*Q*_max_ versus total contact area data for the four thin-film thermoelectric modules of this work. Experimentally measured values are represented by red squares, and the corresponding theoretically predicted values are represented by blue diamonds. The dotted line represents a linear trendline fit to the experimental data. This trendline has been indexed to the origin and is expressed by the equation *y*=601.6*x*.

Figure 6 shows the dependence of maximum cooling power *Q*_max_ as a function of the total element contact area *A*_c_ (including both n and p contacts). The top header area *A*_T_ is 600 × 600 μm^2^ for all of the modules tested. The packing fraction is the ratio of the total element contact area *A*_c_ to the top header area of the module *A*_T_, and the module with a contact diameter of 230 μm has a packing fraction of 48%.

The calculated *Q*_max_ values shown in Fig. 6 are consistently 12–14% larger than what is experimentally observed, which is likely due to other internal parasitic thermal resistances not accounted for in the model shown in equations (4) and (6). Specifically, the internal thermal resistance values that are used in these calculations are obtained using Δ*T*_max_ measurements under the condition of no heat pumping (*Q*_P_=0). In this case the only internal thermal resistance that the measurement is sensitive to is located below the thermoelectric elements, as indicated by *R*_th_ in Fig. 3. However, when heat is being pumped by the module through the top header, as is the case in these heat flux measurements (*Q*_P_>0), any additional thermal resistances, such as thermal spreading resistance that occurs in the module regions above the thermoelectric elements will be important. This additional internal thermal resistance will further reduce the thermoelectric heat pumping in a manner similar to that seen for the lower internal thermal resistance. Capturing the additional internal thermal resistances in the model will reduce the calculated heat pumping and improve agreement between predicted and measured values.

## Discussion

The basis for the larger cooling fluxes produced by thinner thermoelectric elements is the reduced electrical resistance of the thermoelectric structure, which in turn allows for the use of larger electrical currents. The higher currents produce more Seebeck heat pumping per unit area, as shown in equation (8).


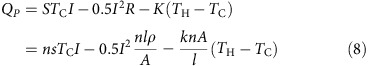


where *Q*_P_ is the amount of heat pumped; *S*, *R* and *K* are the Seebeck coefficient, electrical resistance and thermal conductance of the thermoelectric module, respectively; *T*_C_ and *T*_H_ are the heat source and heat sink temperatures, respectively; *I* is electric current; *n* is the number of thermoelectric couples in the module; *l* is the thickness of one semiconductor leg; *A* is the cross-sectional area of one semiconductor leg (n and p are assumed to have identical geometry); and *s*, *ρl/A* and *kA/l* are the per-couple Seebeck coefficient, electrical resistance and thermal conductance, respectively. Equation (8) does not consider the parasitic thermal and electric resistance in the module.

The input electrical current that corresponds to the case of max *Q*_P_ can be calculated from the first derivative of the *Q*_P_*=f*(*I*) function and is equal to *ST*_C_*/R*. For bulk thermoelectric modules, the maximum value of *Q*_P_ is low, because bulk thermoelectric material cannot be thinned below a thickness of few hundred microns, limiting the maximum current due to the high module electric resistance *R*. The electrical current limitation results in bulk thermoelectric heat-pumping capabilities in the 1–10 W cm^−2^ range.

Epitaxial semiconductor films can be grown much thinner (for example, hundreds of nm), decreasing the electrical resistance and allowing larger electrical currents producing much higher cooling fluxes. For any given reduction in *l* by a factor of *α*, optimized *I* will increase by *α*. With all other things being equal, the cooling flux can be increased by a factor of 2, 5 or 10 times simply by decreasing the film thickness by a factor of 2, 5 or 10 times and increasing the electrical current accordingly. Theoretically, cooling fluxes, on the order of several hundred W cm^−2^, can be achieved simply by thinning the thermoelectric material. However, there are several parasitic effects that need to be overcome to achieve the full potential of thin-film thermoelectric modules. The most significant barrier to high cooling fluxes is electrical contact resistance between metal electrodes and the semiconductor layers. In thin thermoelectric modules, the magnitude of the contact electric resistance can be comparable to the values of the resistance of the thermoelectric element itself, which will increase the total electric resistance of the module and reduce its maximum cooling flux. In this work, a low electric contact resistivity *ρ*_c_ in the range of 1–2 × 10^−6^ Ω cm^2^ has been achieved, using the evaporation of Cr/Ni/Au to fabricate the metal electrodes as well as δ-doped superlattice structures. Further reduction to the order of 10^−8^ Ω cm^2^ is desired for thin-film thermoelectric module with element thickness <2 μm.

Another barrier to wide adoption of thin-film thermoelectric modules is thermal resistances in the thermal management system. For heat flux values of several hundred W cm^−2^, low thermal resistances, on the order of 0.03–0.05 cm^2^ K W^−1^, need to be present on both the hot and cold sides of the thermoelectric module for efficient integration of thermoelectric coolers into the system. In particular, a low thermal resistance present at the hot side takes the higher priority, due to the greater heat flow through the hot side, with *Q*_hot_=*Q*_cold_ (1+1/COP). This requires more advanced heat exchangers and cold plates.

## Methods

### Q-meter method for characterization of thin-film thermoelectric modules

**Figure 7:**
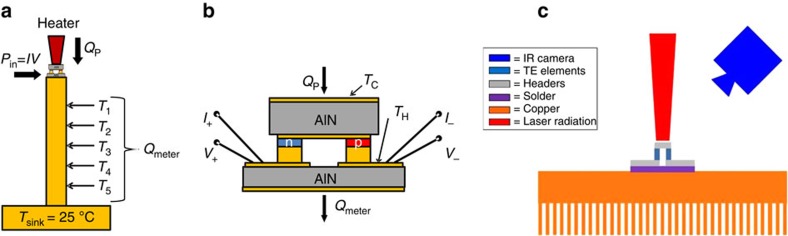
Experimental arrangements for the measurement of heat pumped by the thermoelectric module. (**a**) Schematic of a thermoelectric module mounted on a Q-meter. *T*_1_ through *T*_5_ represent the thermocouple measurements along the length of the Q-meter. (**b**) Schematic of a thermoelectric module showing the heat pumped by the module (*Q*_P_) and additional contributions of the electrical input power (*P*_in_) and wire conduction/heat generation (*P*_wire_) to the heat measured by the Q-meter (*Q*_meter_). The placement of the thermocouples on the cold side and hot side of the thermoelectric module is indicated by *T*_C_ and *T*_H_, respectively. (**c**) Testing apparatus used to perform non-contact measurements of cooling flux *q* and temperature difference Δ*T* of the thermoelectric modules.

To evaluate the heat-pumping capacity, the thermoelectric modules are placed on top of a Q-meter (Fig. 7), a metal post of known dimensions and thermal conductivity, used to determine the total heat flow passing through it by measuring the temperature gradient along its length. The Q-meter base is mounted to a water-cooled heat sink and the thermoelectric module is mounted to the top. The thermoelectric module is energized using a four-wire current–voltage measurement system employing thin Cu wires, approximately 2.5 cm long (either 250 or 500 μm in diameter), to connect the thermoelectric module to external instrumentation. Heat is applied to the top of the thermoelectric module using a resistive heater. The top and bottom thermoelectric module temperatures *T*_C_ and *T*_H_, respectively, are read using 25 μm diameter thermocouples precisely positioned on the thermoelectric module. The entire system is operated under vacuum (*P*<1 mTorr) to minimize convective losses. The water-cooled heat sink on the Q-meter is adjusted to maintain the thermoelectric hot-side temperature (*T*_H_) at about 25 °C. Since the test chamber is evacuated, convective losses are minimized. Thus, the heat flowing down the Q-meter is the sum of heat being pumped by the thermoelectric module, *Q*_P_, the electrical input power flowing into the thermoelectric module, *P*_in_ and the thermal contribution of the four wires connecting the thermoelectric module to the external circuit, *P*_wire_ (*Q*_meter_=*Q*_P_+*P*_in_+*P*_wire_).

### Non-contact method for characterization of thin-film thermoelectric modules

The performance of the thermoelectric modules was also characterized using the non-contact IR method. Figure 7c shows a schematic of the apparatus used for testing, consisting of a heat sink, the thermoelectric module, a laser and an infrared camera. The testing procedure consisted of first providing the thermoelectric module with electrical power, creating a temperature difference across the module. The laser was then used to heat the cold side of the thermoelectric module, decreasing the module-level Δ*T*. The laser power was gradually increased until the Δ*T* across the module was 0 K, indicating that the maximum heat pumping for that current had been reached. The process was repeated at several electrical powers to determine the electrical current that produced the maximum module heat pumping. The output power of the laser was determined using a calibration curve, which was created *in situ*, taking into account all loses in lenses and optical equipment. The emissivity of the cold side of the thermoelectric module was determined using the infrared camera, and from Kirchhoff's law, the absorptivity of the cold side was assumed to be equal to the emissivity. Thus, the total amount of power being pumped through the module could be readily calculated.

### Nomenclature

A Nomenclature table defining all of the variables used in this work is given in Supplementary Note 1.

## Additional information

**How to cite this article:** Bulman, G. *et al*. Superlattice-based thin-film thermoelectric modules with high cooling fluxes. *Nat. Commun.* 7:10302 doi: 10.1038/ncomms10302 (2016).

## Supplementary Material

Supplementary Information Supplementary Note 1

## Figures and Tables

**Table 1:** ZT data from representative p- and n-type thermoelectric materials.

	Electrical resistivity (Ω m)	Seebeck coefficient (μV K^−1^)	Thermal conductivity (W m^−1^ K^−1^)	ZT
p-type Bi_2_Te_3_/Sb_2_Te_3_	1.02e-5	238	1.2	1.4
n-type δ-doped Bi_2_Te_3−*x*_Se_*x*_	1.37e-5	−276	1.1	1.5

**Table 2:** Specific electric contact resistivity, as measured by transmission line model (TLM) technique, for superlattice thermoelectric elements with different structures and metallization.

Sample	Growth information		Contact metal	Contact resistivity			
	Type	Target structure		*R*_s_ (Ω per sq)	*L*_T_ (μm)	*ρ*_C_ (Ω cm^2^)	*ρ*_C_ (s.d.)
A	n	δ-doped n type	Plated Au	1.57	4.20	2.68e-7	6.88e-8
B	p	Bi_2_Te_3_/Sb_2_Te_3_	Plated Au	0.93	12.26	1.36e-6	4.08e-7
C	n	δ-doped n type	Evap Cr/Ni/Au	1.94	7.81	1.16e-6	1.73e-7
D	p	Bi_2_Te_3_/Sb_2_Te_3_	Evap Cr/Ni/Au	1.15	11.74	1.42e-6	7.95e-7

Evap, evaporated.

**Table 3:** Performance of thin-film thermoelectric modules of varying contact diameter (that is, packing fraction).

Diameter (μm)	Δ*T*_max_ (K)	*I*_max_ (A)	*Q*_max_/*A* (W cm^−2^)			Coefficient of performance
			Predicted	Measured	% Difference	
130	45.58	9.91	183.3	158.3	−13.6	0.86
180	40.49	11.99	213.9	184.1	−13.9	0.40
200	36.06	14.32	247.2	213.7	−13.6	0.72
230	43.54	14.78	294.4	257.6	−12.5	0.51

## References

[b1] Chowdhury I. . On-chip cooling by superlattice-based thin-film thermoelectrics. Nat. Nanotech. 4, 235–238 (2009). 10.1038/nnano.2008.417 19350033

[b2] Mahajan R. . Cooling a microprocessor chip. Proc. IEEE 94, 1476–1486 (2006).

[b3] Prasher R. S. . Nano and micro technology-based next-generation package-level cooling solutions. Intel Tech. J. 9, 285–296 (2005).

[b4] Meysenc L. . Power electronics cooling effectiveness versus thermal inertia. IEEE Trans. Power Electron. 20, 687–693 (2005).

[b5] Joo Goh T. . Thermal investigations of microelectronic chip with non-uniform power distribution: temperature prediction and thermal placement design optimization. Microelectron. Int. 21, 29–43 (2004).

[b6] Semenyuk V. Miniature thermoelectric modules with increased cooling power. Rotter M. (Ed.) in Proc. ICT'06 25th International Conference 322–326 (2006).

[b7] Kim S. . Dense dislocation arrays embedded in grain boundaries for high-performance bulk thermoelectrics. Science 348, 109–114 (2015). 25838382 10.1126/science.aaa4166

[b8] Poudel B. . High-thermoelectric performance of nanostructured bismuth antimony telluride bulk alloys. Science 320, 634–638 (2008). 18356488 10.1126/science.1156446

[b9] Biswas K. . High-performance bulk thermoelectrics with all-scale hierarchical architectures. Nature 489, 414–418 (2012). 22996556 10.1038/nature11439

[b10] Bell L. Cooling heating, generating power, and recovering waste heat with thermoelectric systems. Science 321, 1457–1461 (2008). 18787160 10.1126/science.1158899

[b11] Heremans J. P. . Enhancement of thermoelectric efficiency in PbTe by distortion of the electronic density of states. Science 321, 554–557 (2008). 18653890 10.1126/science.1159725

[b12] Majumdar A. Thermoelectricity in semiconductor nanostructures. Science 303, 777–778 (2004). 14764859 10.1126/science.1093164

[b13] Hsu K. F. . Cubic AgPbmSbTe_2+m_: bulk thermoelectric materials with high figure of merit. Science 303, 818–821 (2004). 14764873 10.1126/science.1092963

[b14] Harman T. C. . Quantum dot superlattice thermoelectric materials and devices. Science 297, 2229–2232 (2002). 12351781 10.1126/science.1072886

[b15] Chung D. Y. . CsBi_4_Te_6_: a high-performance thermoelectric material for low-temperature applications. Science 287, 1024–1027 (2000). 10669411 10.1126/science.287.5455.1024

[b16] Rowe D. M. (ed.) Thermoelectrics Handbook: Macro to Nano CRC Press (2005).

[b17] Yang B. & Wang P. Encyclopedia of Thermal Packaging, Volume 4: Thermoelectric Microcoolers World Scientific (2013).

[b18] Venkatasubramanian R. . Thin-film thermoelectric devices with high room-temperature figures of merit. Nature 413, 597–602 (2001). 11595940 10.1038/35098012

[b19] Jood P. . Al-doped zinc oxide nanocomposites with enhanced thermoelectric properties. Nano Lett. 11, 4337–4342 (2011). 21910447 10.1021/nl202439h

[b20] Chen X. . Effects of ball milling on microstructures and thermoelectric properties of higher manganese silicides. J. Alloys Compd. 641, 30–36 (2015).

[b21] Kraemer D. . High-performance flat-panel solar thermoelectric generators with high thermal concentration. Nat. Mater. 10, 532–538 (2011). 21532584 10.1038/nmat3013

[b22] Majumdar A. Thermoelectric devices: helping chips to keep their cool. Nat. Nanotechnol. 4, 214–215 (2009). 19350027 10.1038/nnano.2009.65

[b23] Su X. L. . Self-propagating high-temperature synthesis for compound thermoelectrics and new criterion for combustion processing. Nat. Commun. 5, 4908 (2014). 25223333 10.1038/ncomms5908 PMC4175591

[b24] Wu H. J. . Broad temperature plateau for thermoelectric figure of merit ZT > 2 in phase-separated PbTe_0.7_S_0.3_. Nat. Commun. 5, 4515 (2014). 25072798 10.1038/ncomms5515

[b25] Shakouri A. Nanoscale thermal transport and microrefrigerators on a chip. Proc. IEEE 94, 1613–1638 (2006).

[b26] Zhang Y. . in ASME 2005 Pacific Rim Technical Conference and Exhibition on Integration and Packaging of MEMS, NEMS, and Electronic Systems collocated with the ASME 2005 Heat Transfer Summer Conference 2189–2197 (2005).

[b27] Rowe D. M. (ed.) CRC Handbook of Thermoelectrics CRC Press (1995).

[b28] Bulman G. E. . Three-stage thin-film superlattice thermoelectric multistage microcoolers with a ΔT max of 102 K. J. Electron. Mater. 38, 1510–1515 (2009).

[b29] Habbe B. & Nurnus J. Thin film thermoelectrics today and tomorrow. Electron. Cooling 17, 24–31 (2011).

[b30] Wang P., Yang B. & Bar-Cohen A. Mini-contact enhanced thermoelectric coolers for on-chip hot spot cooling. Heat Transfer Eng. 30, 736–743 (2009).

[b31] Yang B. . Mini-contact enhanced thermoelectric cooling of hot spots in high power devices. IEEE Trans. Compon. Packag. Technol. 30, 432–438 (2007).

[b32] Lents C. E. . DARPA ACM program final report. Contract No. N66001-11-C-4053 (2013).

[b33] Schroder D. Semiconductor Material and Device Characterization Wiley-IEEE Press (2015).

[b34] Bulman G. . Large external ΔT and cooling power densities in thin-film Bi_2_Te_3_-superlattice thermoelectric cooling devices. Appl. Phys. Lett. 89, 122117 (2006).

[b35] Nolas G. S., Sharp J. & Goldsmid H. J. Thermoelectrics: Basic Principles and New Materials Developments Springer (2001).

