# Supplementary material for: Superlattice-based thin-film thermoelectric modules with high cooling fluxes
Source: Nat Commun. 2016 Jan 13;7:10302. doi: 10.1038/ncomms10302 (PMC4735511; doi:10.1038/ncomms10302)
Supplement: Supplementary Information — Supplementary Note 1 [file ncomms10302-s1.pdf]

SUPPLEMENTARY NOTE 1

Nomenclature

A_C	Total element contact area, m^2
A_T	Top header area of thermoelectric module, m^2
d	Transmission line measurement gap width, m
f	Packing fraction
I	Electric current, A
K	Thermal conductance, W/K
l	Thickness of thermoelectric element, m
L_T	Transmission line measurement transfer length, m
n	Number of thermoelectric couples
P	Pressure, torr
Q_P	Cooling power, W
q_{max}	Maximum cooling flux, W/m^2
R	Electrical resistance, Ω
R_{th}	Parasitic thermal resistance, K/W
S	Seebeck coefficient, V/K
T	Absolute temperature, K
V	Voltage, V
ZT	Dimensionless thermoelectric figure of merit
ΔT	Temperature difference, K
κ	Thermal conductivity, W/m-K
ρ	Electrical resistivity, Ω-m
ρ_C	Specific contact resistivity, $\Omega\text{-}m^2$
